# Japan

**Published:** 1898-05

**Authors:** 


JAPAN has almost 70,000,000 of people, and 31,000 physicians. There are eight schools of medicine in Japan; that is to say, that the medical art is not on the decline in the race that has been called the French of the Orient. We all know how learned Japanese doctors are. There, for instance, is Kitasato, who first isolated and cultivated the bacillus of tetanus. As to the practical part of the healing art, we know but little, but they have good schools, especially at Tokio.

